# Determinants of retention in HIV antiretroviral treatment (ART) in the Cameroon International epidemiology Database to Evaluate AIDS (IeDEA) study clinics: the context of the HIV treat all strategy in Cameroon

**DOI:** 10.11604/pamj.2021.40.129.22642

**Published:** 2021-11-02

**Authors:** Rogers Awoh Ajeh, Halle Ekane Gregory, Egbe Obinchemti Thomas, Nsah Awachwi Noela, Anastase Dzudie, Assob Nguedia Jules, Adebola Adedimeji

**Affiliations:** 1 Faculty of Health Sciences, University of Buea, Buea, Cameroon,; 2 Clinical Research Education Networking and Consultancy, Yaoundé, Cameroon,; 3 Integrated Research Group, Yaoundé, Cameroon,; 4 Albert Einstein College of Medicine, New York, United States

**Keywords:** Determinants, antiretroviral treatment, retention, HIV-treat-all, Cameroon

## Abstract

**Introduction:**

retaining patients in antiretroviral treatment (ART) is essential for successful outcomes. Unfortunately, Cameroon continues to report suboptimal ART retention. This study focused on identifying determinants of ART retention in three HIV clinics in Cameroon within the HIV treat all context.

**Methods:**

a medical chart review of 423 subjects who initiated ART between July and September 2016 in the Limbe, Bamenda and Jamot Hospitals. Patients' sociodemographic and clinical characteristics and ART retention data were abstracted using structured paper forms. Chi square test was used to assess bivariate associations. Logistic regression was used to adjust for confounders. P-value was set at <0.05 at 95% confidence interval.

**Results:**

the mean age was 40±11 years, and 65.1% were females. Antiretroviral treatment retention after 24 months was 309/392 (78.83%) and 30/423 (7.1%) were transferred-out, 11/423 (2.6%) reported dead and 73/423 (17.3%) lost to follow-up. HIV status disclosure (AOR 0.16 95% CI: 0.05-0.51, p<0.01) and age group 31-50 years (AOR 3.63, 95% CI: 1.04-12.59, P= 0.04) were associated with lower and higher ART retention respectively.

**Conclusion:**

about a quarter of the participants were not retained in ART after 24 months. Patient-level factors determined ART retention. These factors should be considered in designing strategies to improve ART retention. More research is needed to identify other determinants of ART retention under the HIV treat all strategy.

## Introduction

HIV/AIDS remains a major public health problem with the highest burden of the disease in sub-Saharan Africa [1]. In 2018, United Nations Programme on HIV (UNAIDS) reported 37.9 million people were living with HIV with 1.7 million new cases of HIV infection [2]. However, remarkable expansion of access to ART has given 21.7 million adults living with HIV the opportunity to achieve increased life expectancy [1,2]. ART has been shown to be effective in reducing mortality and onward transmission among those who remain in treatment and adhere to therapy and thus achieve viral suppression [3,4]. It has been demonstrated that there is a marked reduction in morbidity and mortality rates among persons living with HIV (PLWH) who initiate ART at CD4 counts greater than 500/mm^3^ compared with individuals starting therapy at CD4+ counts below 350/mm^3^ [5,6]. In 2015, WHO recommended the HIV test and treat strategy as a global approach for the control of the HIV/AIDS´ pandemic. The universal strategy aimed at increasing antiretroviral therapy (ART) coverage through testing everyone for HIV, initiating those tested HIV positive on ART irrespective of CD4 count or clinical criteria and maintaining PLWH on ART in order to achieve sustained viral suppression.

With ambition to attain the triple 90 target, Cameroon, in May 2016, implemented the World Health Organization (WHO) recommended HIV “Test and treat” strategy in all health facilities providing HIV services within the national territory [7]. In 2017,77,218 PLWH were newly initiated on ART in Cameroon, an increase by 18 % from a total of 65,194 in 2016. At the end of 2017, 49% of the 510,000 PLWH in Cameroon were on ART with a reported retention rate of 78% and 12,000 deaths averted in that same year [2]. Retaining people living with HIV in ART and care is an essential prerequisite to attaining the overall goal of the HIV test and treat strategy triple 90 target by 2020, and thus ending the pandemic by 2030. However, ART retention has remained sub optimal in many sub-Saharan African countries, including Cameroon [8]. Furthermore, trends toward an increased risk of early lost to follow-up have been associated with the HIV test and treat strategy [9], and there´s very limited literature on the determinants of retention under the HIV test and treat guidelines. Identifying factors associated with ART retention in care under this strategy may be helpful in improving ART retention outcomes. This study aimed at identifying determinants of ART retention after 24 months in three tertiary hospitals´ HIV clinics in Cameroon.

## Methods

**Study design and setting:** we conducted a retrospective study, which constituted a hospital-based 24-month follow-up of the clinic records of all eligible patients who initiated ART from July to September 2016 in three Cameroon-based HIV clinics. The study was carried out within the framework of a larger study, the International Epidemiology database to evaluate AIDS (IeDEA), Cameroon. The study was thus implemented in all the three active clinics of the IeDEA Cameroon study. The study sites included: the Bamenda Regional Hospital (BRH) in the North West Region, the Limbe Regional Hospital (LRH) in the South West Region, and the Yaoundé Jamot Hospital (YJH) in the Centre Region of Cameroon. All the three sites are tertiary level public service facilities, with specialized HIV treatment centers and provided care to over 12,500 HIV positive clients; BRH (5000), LRH (4000) and YJH (3500). Each of the HIV clinics had a medical doctor in charge of the treatment center, and other staff including nurses, pharmacy attendants, counsellors and psychosocial workers.

**Figure 1:**
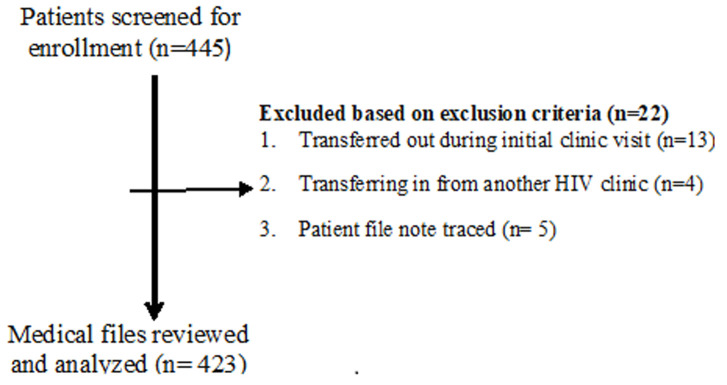
participants' recruitment procedure

**Study participants, inclusion and exclusion criteria:** eligible study participants included all HIV positive adult patients, aged 19 and above, enrolled in care in the three study clinics within the study reporting period (July to September 2016). Patients with evidence of enrollment (registered in the ART enrollment registers), whose medical files could not be traced were excluded from the study. Patients transferred out to other clinics during their first visit were excluded. Patients transferred in from other clinics were also excluded. Participants´ recruitment is schematically illustrated in Figure 1.

**Variables:** patients´ medical records, in accordance with an exhaustive list derived chronologically according to date of registration in the clinic ART register during the study reporting period, were sorted and data abstracted in each of the three study clinics. A structured form that was pre tested on 30 medical files (10 per study clinic), was used to abstract socio demographic, clinical, ART retention data from the patients´ medical records in the three study clinics.

### The variables measured are detailed below

**Sociodemographic variables:** age group, sex, level of education, marital status, monthly income, travel distance from patients´ home to HIV clinic, alcohol consumption, cigarette smoking and HIV clinic.

**Clinical variables:** CD4 count and HIV status disclosure.

**Antiretroviral treatment retention:** proportion of subjects who initiated ART known to have accessed ART in the study clinics during the 24-month study follow up period.

**Transferred out:** proportion of patients with clinic level documented evidence of being transferred from the study clinic to another HIV clinic.

**Lost to follow up:** proportion of patients who failed to return for drug pick-up after three consecutive months following the last expected pharmacy appointment date, and were reported as lost to follow up in the clinic ART registers.

**Deceased:** proportion of patients with clinic level documented evidence of being deceased.

**Data sources and measurements:** data for the study were obtained exclusively from the patients´ medical charts and ART registers in the respective HIV clinics. It may be worth noting that all the three HIV clinics used a standard patients´ medical chart and ART register provided by the Cameroon National AIDS control program all the HIV clinics within the National Territory. Antiretroviral treatment retention was measured after 24 months following ART initiation in the study site.

**Bias:** to minimized sampling bias, we included all eligible patients enrolled in ART and care within the study reporting period. A harmonized data collection tool was used to collect data across the three sites by three well-trained and closely monitored research assistants to minimize data collection biases. Furthermore, rigorous data quality assurance protocols were device for data abstraction from medical records across the three sites. Data quality control was done on the central data base and inconsistencies verified at the study clinics and corrected. Data quality control measures included a complete data source verification of 20% of the completed paper forms per site, progressive trouble shooting of database to identify outliers, inconsistences and double entries, and corrections made. Abstracted paper forms were safeguarded in a locked cupboard after entry and the database backed up in an external hard drive.

**Statistical methods:** the data was analysed using Epi Info version 7 software. Means and standard deviations were calculated for continuous variables (age) while frequency distributions calculated for categorical variables (age group, sex, marital status, education level, monthly income, travel distance, HIV status disclosure, CD4 group, alcohol consumption, cigarette smoking and HIV clinic site). Comparison of proportions were done using Chi square. A multivariate logistic regression was used to assess for independent associations for covariates that were significant at the bivariate analysis, or had p-values < 0.2 were included. Age and sex were systematically included in the model. Statistical significance was considered at p value <0.05 at 95% confidence interval.

**Ethical considerations:** an ethical clearance was obtained from the Faculty of Health Science Institutional Review Board of the University of Buea, Ref: 2018/316-05/UB/SG/IRB/FHS [10] prior to the implementation of the study. Consent waiver was obtained for this study, as it constituted an exclusively secondary data collection process, with no direct interaction with patients. No direct patient identifying information was captured during the entire data collection process. Ethical and good clinical practice principles were respected at all levels of the study implementation.

## Results

Four hundred and forty five (445) potentially eligible patients were screened for medical chart review. Twenty-two (22) patients were excluded on bases of the study exclusion criteria: transferred out during initial clinic visit (13), transferred in (04) and missing patients files (05). In the end, data was abstracted from 423 patients´ records including 112 from Limbe, 113 from Bamenda and 198 from Jamot. The mean age (years) of the subjects was 39.72±8. About 232/408 (57%) had at least a secondary level of education and only 23/216 (10.7%) had an income >100,000FCFA ($190). Slightly less than half 172/400 (43%) of the patients were unmarried and just 20% were employees. More than half 193/345(56%) of the patients lived within 30 minutes from the HIV clinic and up to 297/376(79%) had disclosed their HIV status to someone. Regarding substance use, 231/353(65%) of patients reported they consumed alcohol and majority 340/415(82%) had never smoked cigarette. Table 1 summarizes the socio-demographic characteristics of the subjects.

**Table 1:** socio-demographic characteristics of the study participants

Variable	Numbers (N)	Percentages (%)
**Age in years, N=412**		
19-30	89	21.60
31-40	146	35.44
41-50	108	26.21
>50	69	16.75
**Gender, N=423**		
Male	148	34.92
Female	275	65.08
**Marital status, N=400**		
Married	152	38.00
Unmarried	172	43.00
Divorced	51	12.75
Widowed	25	6.25
**Employment status, N=397**		
Employed by an institution	81	20.40
Self-employed	171	43.07
Unemployed	145	36.52
**Income (FCFA), N=216**		
<50,000	148	68.52
50-100000	45	20.83
>100,000	23	10.65
**Level of education, N=408**		
None	29	7.11
Primary	147	36.03
Secondary	199	48.77
University	33	8.09
**Home to clinic travel duration, N=345**		
0-30 minutes	193	55.94
0.5-1 hour	98	28.14
1-2 hours	34	9.86
>2hours	20	5.80
**Alcohol, N=353** No	122	34.56
Yes	231	65.44
**Smoking, N=415**		
Yes	75	18.68
No	340	81.32
**HIV status Disclosure, N=376**		
Yes	79	21.01
No	297	78.99

**HIV clinical characteristics:** the median CD4 cell count value was 225 (101-411) cells/mm^3^ at initiation and 518 (240-550) cells/mm^3^ at 24 months. Similarly, the mean weight increased from 66±15kg at initiation to 72±17kg at 24 month. The mean systolic and diastolic blood pressure (BP) values at initiation and 24 months were 129±24,124±17 and 75±16, 81±14mmHg respectively.

**ART retention:** at the end of the 24 month, 30/423 (7.1%) were transferred out, 11/423 (2.6%) were reported dead, 73/423 (17.3%) were lost to follow up and 309/432 (73.0%) remained in care. Antiretroviral treatment retention rate at 24 months was 309/392(78.83%). The ART retention outcomes are described in Table 2.

**Table 2:** multivariate associations with ART retention

	ART Retention					
	No	No (%)	OR (CI)	P-value	AOR (CI)	P-value
**Age in years (n=382)**						
19-30	82	66 (80.49)	1.41 (0.63-3.13)	0.404	1.52 (0.4-5.83)	0.539
31-40	136	109(80.15)	1.38 (0.67-2.83)	0.386	3.63 (1.04-12.59)	**0.043**
41-50	105	80(76.19)	1.09 (0.52-2.28)	0.817	2.11 (0.63-7.11)	0.227
>50	59	44(74.58)	1		1	
**Sex (n=390)**						
Male	139	106(76.26)	0.80 (0.49-1.32)	0.378	0.81 (0.32-2.04)	0.656
Female	251	201(80.08)	1		1	
**Employment (n=368)**						
Self-employed	162	121(74.69)	0.35 (0.15-0.78)	0.011	0.31 (0.08-1.14)	0.077
Unemployed	130	103(79.23)	0.45 (0.19-1.05)	0.064	0.74 (0.18-3.07)	0.676
Employed by institution	76	68(89.47)	1		1	
Income in FCFA (n=301)						
>100000	21	19(90.48)	3.08 (0.70-13.62)	0.138	2.06 (0.33-12.75)	0.439
50000-100000	43	37(86.05)	2.00 (0.80-4.97)	0.137	2.11 (0.46-9.74)	0.341
<50000	237	179(75.53)	1		1	
Marital status (n=370)						
Married	143	119(83.22)	1.65 (0.59-4.60)	0.336	1.25 (0.3-5.24)	0.758
Unmarried	157	115(73.25)	0.91 (0.34-2.45)	0.856	0.77 (0.19-3.15)	0.717
Widowed	46	42(91.30)	3.50 (0.88-13.92)	0.075	6.27 (0.91-43.26)	0.062
Divorced	24	18(75)	1		1	
Level of education (n=377)						
University	31	28(90.32)	1.78 (0.36-8.81)	0.481		
Secondary	187	143(76.47)	0.62 (0.20-1.90)	0.402		
Primary	134	107(79.85)	0.76 (0.24-2.38)	0.632		
None	25	21(84)	1			
Time of travel (n=314)						
0-30minutes	178	139(78.09)	1.02 (0.32-3.27)	0.976		
05-1 hour	86	59(68.60)	0.62 (0.19-2.08)	0.442		
1-2 hours	32	28(87.50)	2.00 (0.43-9.21)	0.374		
>2hours	18	14(77.78)	1			
Alcohol (n=314)						
Yes	193	159(82.38)	1.98 (1.16-3.39)	0.013	1.14 (0.52-2.51)	0.744
No	121	85(70.25)	1		1	
Smoking (n=392)						
Yes	71	46(64.79)	0.41 (0.23-0.71)	0.002	0.56 (0.2-1.53)	0.256
No	321	263(81.93)	1		1	
HIV Status disclosure (n=346)						
Yes	270	206(76.30)	0.33 (0.14-0.75)	0.008	0.16 (0.05-0.51)	0.002
No	76	69(90.79)	1		1	
Study clinic (n=392)						
Limbe	100	74(74)	0.32 (0.15-0.69)	0.004	0.66 (0.21-2.22)	0.503
Jamot	183	137(74.86)	0.33 (0.17-0.68)	0.002	0.43 (0.14-1.37)	0.153
Bamenda	109	98(89.91)	1		1	
CD4 at initiation (n=372)						
>200cells/ml	200	165(82.50)	1.67 (1.02-2.75)	0.044	1.74 (0.82-3.72)	0.152
<200cells/ml	172	127(73.84)	1		1	

OR- odds Ratio CI- confidence interval

**Bivariate associations with ART retention after 24 month:** antiretroviral treatment retention decreased with increasing age group, from 80.5% at 19-30 years through 76.2% at 31-50, to 74.58% at >50 years. However, the association was not significant (p=0.60). Retention was higher amongst the women compared to the men, though this was also not statistically significant (80.08% vs. 76.26% p=0.37). Subjects who were formal employees were significantly (p=0.02) more likely to be retained (89%) compared to the unemployed and self-employed (79% and 74%). And about 111/140 (80%) of the self-employed had a monthly income of less than $83 per month, while about 105/113 (93%) of unemployed had no regular income. Suggesting lowest monthly incomes amongst self-employed and unemployed category. Regarding marital status, widows/widowers had a significantly (P=0.02) higher ART retention (91.30%) compared with the unmarried (73.25%) and the divorced (75.0%). About 42/51 (82%) of the widowed were female. Slightly over 120/170 (70%) of the unmarried and 20/25 (80%) of divorced were females.

Educational level was not associated with ART retention though the retention was highest amongst those with university education (90.32%). There was also no significant association between the time of travel to the clinic and ART retention though the retention rate was highest (87.50%) amongst those who lived 1-2 hours away from the clinic. Alcohol consumption was significantly associated with higher retention (82.38 vs. 70.25, p=0.01). This association was however not significant after adjusting for confounders. Similarly, smoking cigarette was also significantly associated with lower retention at the bivariate level but not significant after adjusting for confounders. Disclosure of HIV status was adversely related to ART retention, with those who had disclosed their HIV status having lower retention proportion compared with those who had not disclosed status (76.30% vs. 90.79%, p<0.01). CD4 count greater than 200cells/µl at initiation was associated with higher retention compared to those with a CD4 count less than 200cells/µl (82.50% vs 73.84% p= 0.04). ART retention varied with HIV clinic, with Limbe (74.0%) and Jamot (74.86%) clinics having lower ART retention compared with Bamenda (74.01% and 74.86% vs. 89.91, p<0.01) Table 2 summarizes the bivariate associations with ART retention.

**Multivariate associations with ART retention:** after adjusting for confounders, participants of the age group 31-40 (years) were associated with higher odds of being retained in ART (AOR 3.63, 95%CI1.04-12.95, P=0.04) compared with the older age group >50 years. HIV status disclosure was associated with lower odds of being retained in ART (AOR 0.16 95% CI 0.05-0.51, p<0.01). Table V summarizes the multivariate associations with ART retention.

## Discussion

ART retention and associated factors have not been extensively reported following the implementation of the HIV test and treat (T&T) strategy in cameroon in 2016. Determinants of ART retention may change with changing ART guidelines and over time. This study assessed ART retention at 24 months and its associated factors amongst patients initiated in care under the HIV T&T guidelines in three Cameroon HIV clinics. The study found a general 24 months ART retention proportion of 78%. Age group and HIV disclosure status were associated with ART retention after 24 months. An up to date knowledge of ART retention and its determinants is needed to inform strategies to ensure optimal ART retentions outcomes within the HIV test and treat context in Cameroon.The observed decline in ART retention over the 24 months study period is typical of ART programs, with a decreasing proportion of patients remaining in care over time [3,11]. The 24 month ART retention (79%) reported by this study is higher than 12 months retention (60%) reported by a Cameroon national survey carried out in 2014 and retention after 24 months (73%) in 2016 [8,12] and similar to that reported in 2017 (81%) in the South West Region of Cameroon [13]. The trends suggest some improvement in ART retention over time, though the trends still remain suboptimal with referenced to the WHO recommended retention 90% target by 2020. The improvement could be due to general increase in HIV awareness and stigma reduction especially following the nationwide implementation of the HIV test and treat strategy in Cameroon. Intensification of the activities of psychosocial workers in the search and re-engagement of patients lost to follow up (LTF) following the implementation of the HIV test and treat strategy in Cameroon could also be a factor contributing to the improvement. We noted that all the three clinics in this study have state employed psychosocial workers with primary responsibility to identify and reengage patients LTF back in care.

It has been reported that more that 75% of patient LTF could be traced and a good proportion return to ART and care at some point [14,15]. Regarding the determinants of ART retention, the observed higher retention in age group (31-40 years) is consistent with the findings from other studies, which have shown higher retention with older age groups compared with lower age groups [16,17]. However, the decreased odds of being retained in the oldest age group (>50 years) in this study could be suggestive of higher undocumented mortality. Undocumented mortality is common in ART programs and contributes significantly to the proportion of patients not retained in ART [18,19]. Moreover, higher specific mortality rates have been reported amongst people living with HIV compared to the general population [20,21]. A cohort study in Uganda reported nearly twice higher risk of mortality amongst older adults (50 years and older) comparted with HIV-negative adults [22]. More targeted studies are needed to understand the causes of non-retention in older patients in ART, in order to inform interventions to improve retention in this group.

The inverse association between HIV status disclosure and ART retention reported in this study is contrary to what we expected. A similar association was reported in Nairobi in 2018 [11] and in Cameroon in 2019 [12]. It could be understandable that some HIV infected persons who have not disclosed their HIV status could become highly motivated to physically appear very healthy in order to conceal their serologic status to avoid HIV related stigma and discrimination. Remaining in care and adhering to ART have been reported to be measures taken to remain healthy and appear uninfected by some HIV infected persons [23]. In an unpublished study carried out in the South west region of Cameroon, more than 80% of the participants expressed regret disclosing their HIV status to others. HIV status disclosure to the wrong person may attract HIV related stigma and adversely influence ART retention. Healthy and independent individuals may not need much psychosocial support from informal care givers and may rather attract avoidable stigma resulting from HIV status disclosure. However, many studies have reported rather positive associations between HIV status disclosure and ART retention [24,25]. It is worth noting that the studies which reported an inverse association between HIV status disclosure and ART retention are relatively more recent, and carried within the HIV test and treat context. This suggests that context and consequences of HIV status disclosure may change with changing ART guidelines. More research is needed to better understand the dynamics and influence of HIV status disclosure on ART retention.

**Limitations of the study:**this study was subjected to some the traditional limitations associated with retrospective chart reviews. A set of measures were however put in place to minimize the limitations while increasing the strength. An explicit protocol and well-defined inclusion and exclusion criteria were used and data abstracted from all the medical charts meeting the study inclusion criteria to minimized risk of selection bias. Standardized piloted abstraction forms were used and the variables to be collected were well defined in order to minimize misclassification bias. The data was abstracted across the three study sites increasing the representativeness of the study. Records with missing data on the outcome variables were not included in the analysis and this did not affect the results of the study as there were very few missing records which were random.

## Conclusion

About a quarter of the patients enrolled in ART were not retained after 24 months. This suggests that Cameroon is still lagging behind the UNAIDS recommended target of attaining 90% ART retention. Patient level factors including age and HIV status disclosure should be taken into consideration when designing strategies improve retention in ART programs. Targeted research studies are needed to uncover other underlying factors, identify and test innovative strategies to improve ART retention within the HIV universal test and treat context.

### What is known about this topic

- The determinants of ART retention have been reported by some previous studies in Cameroon, mainly under the pre-HIV treat all guidelines.

### What this study adds

- The determinants of ART retention under the HIV treat all guidelines have not been extensively reported in sub-Saharan Africa countries;
- The authors did not come across any published article reporting determinants of ART retention amongst patients enrolled under the HIV treat all context in Cameroon.
